# Male infertility and the risk of developing prostate cancer: a bidirectional two-sample Mendelian randomization study

**DOI:** 10.1186/s40001-025-03461-y

**Published:** 2025-12-01

**Authors:** Junjie Shao, Aireti Apizi, Xiaojie Zheng, Ning Tao, Hengqing An

**Affiliations:** 1 Department of Urology, The First Affiliated Hospital of Xinjiang Medical University, Urumqi, China; 2 Research Management Section, The First Affiliated Hospital of Xinjiang Medical University, Urumqi, China; 3 School of Public Health, Xinjiang Medical University, Urumqi, China; 4 Xinjiang Clinical Research Center for Urological and Andrological Diseases, Urumqi, China

**Keywords:** Male infertility, Prostate cancer, Mendelian randomization, Genome-wide association study, Inverse variance weighted

## Abstract

**Background:**

Although observational studies have suggested an association between male infertility and prostate cancer (PCa) risk, the causal relationship has not been established.

**Methods:**

We conducted a bidirectional two-sample Mendelian randomization (MR) analysis using genome-wide association study (GWAS) summary data to investigate the causal relationship between male infertility and PCa. The primary analysis was performed using the inverse variance weighted (IVW) method, supplemented by three additional MR approaches. We systematically evaluated the heterogeneity and pleiotropy of the instrumental variables and employed linkage disequilibrium score regression (LDSC) to assess the genetic correlation between the two conditions. Furthermore, we incorporated factors such as obesity and prostatitis into a multivariable Mendelian randomization framework. Additionally, within the bidirectional MR framework, we explored the causal association between the expression of the PCa biomarker prostate-specific antigen (PSA) and male infertility. Finally, we employed a summary data-based Mendelian randomization (SMR) analysis to identify genes potentially involved in the pathogenesis of PCa. We then performed a preliminary analysis of the differential expression of these significant candidate genes between normal and cancerous tissues using publicly available databases.

**Results:**

Based on the fundamental principles of MR, six instrumental variables for male infertility were selected for analysis with PCa. All four analytical methods (primarily the IVW method, OR = 1.0044, 95% CI = 0.9824–1.0269, *P* = 0.697) consistently indicated the absence of a causal association. Reverse MR analysis suggested no significant causal relationship (IVW, OR = 0.9769, 95% CI = 0.85–1.1123, *P* = 0.724). Sensitivity analyses, including heterogeneity tests (Cochran’s Q), pleiotropy assessment (MR-Egger intercept), and leave-one-out validation, confirmed the robustness of these findings. Furthermore, LDSC indicated no significant genetic correlation between male infertility and PCa (Rg = − 0.1102, *P* = 0.494). After excluding the influence of other exposure factors, multivariable Mendelian randomization results suggested an independent causal effect of prostatitis on PCa risk (IVW, OR = 1.093, 95% CI = 1.0206–1.1707, *P* = 0.011). No significant causal association was demonstrated between PSA and male infertility (IVW, OR = 0.981, 95% CI = 0.8055–1.1942, *P* = 0.847).

**Conclusion:**

Our findings provide no evidence for a causal relationship between male infertility and PCa in either direction.

**Supplementary Information:**

The online version contains supplementary material available at 10.1186/s40001-025-03461-y.

## Introduction

Infertility is defined as the failure to conceive after one year of regular, unprotected sexual intercourse. From 1950 to 2017, the global fertility rate decreased from 4.7 to 2.4. Approximately 15% of couples are affected by infertility, with male factors contributing to roughly half of these cases. Between 1981 and 2013, male sperm counts exhibited a persistent decline, averaging an annual reduction of 0.7 million/ml [1]. Male infertility is regarded as a barometer of men’s health. Mounting evidence indicates a correlation between male infertility status and the prevalence of various diseases, including oncological, cardiovascular, autoimmune, and other chronic conditions [2].

Prostate cancer (PCa) ranks as the second most prevalent malignancy among men and the fifth leading cause of cancer-related mortality [3]. In the United States alone, approximately 3.3 million men have been diagnosed with PCa [4]. As the global population continues to age, PCa has emerged as the most prevalent cancer in men in the majority of countries worldwide (112 out of 185). Annually, there are over 1.2 million new PCa cases and over 350,000 PCa-related deaths [5]. By 2040, the mortality rate associated with metastatic PCa is projected to more than double [6]. Age, ethnicity, and family history are established nonmodifiable risk factors for PCa. Metabolic syndrome, obesity, and cigarette smoking have been identified as potential modifiable risk factors. In the pursuit of optimized early screening strategies for PCa, ongoing evaluations are being conducted to identify additional potential risk factors. A case–control study in Sweden indicates that male infertility is linked to a lower risk of PCa [7]. Nevertheless, a systematic meta-analysis found no definitive association between these two conditions [8]. Contradictorily, an analysis spanning 20 years of the entire Swedish population implies that male infertility could be associated with an increased PCa risk, yet a causal relationship remains to be established [9].

In the realm of research, controversial issues are often of great importance. When trying to demonstrate a causal relationship between two diseases, randomized controlled trials (RCTs) are considered gold standards for identifying causality between exposure and outcome. However, due to various limitations, RCTs are not easily implemented in this context. With the development of genetic epidemiology, Mendelian randomization (MR) has emerged as a method increasingly used to infer the causal effects of exposures on outcomes by using genetic variants. MR has several advantages over observational studies. First, as alleles are randomly assorted during meiosis, using genetic variants as instrumental variables (IVs) minimizes residual confounding of exposure factors related to the outcome. Second, an individual’s genotype is determined at fertilization, making reverse causation, where the disease alters genotype, highly unlikely. Third, with sufficient sample sizes, IVs estimates are not attenuated by measurement errors in exposure factors, including intra-individual variability, a common issue in classical observational studies [10]. In this study, we performed bidirectional MR analysis using two independent samples to determine if a specific causal relationship exists between male infertility and PCa.

## Materials and methods

### Study design and data sources

**Fig. 1:**
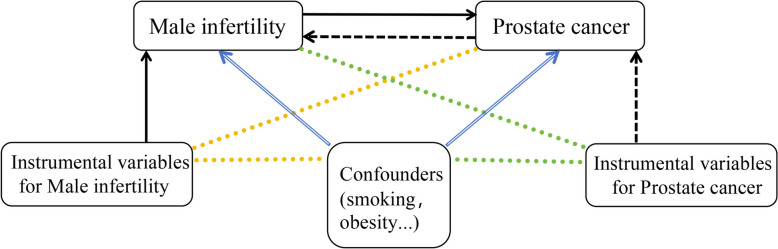
Directed acyclic graph (DAG) of the study design

The present study involved a two-sample MR analysis of two independent samples to explore whether a causal relationship exists between male infertility status and the development of PCa (Fig. 1). The data used in the study were all from publicly available genome-wide association studies (GWAS), with IVs data for male infertility obtained from FinnGen (https://www.finngen.fi/en/), whose investigators collect and analyze genomic and health data from donors to the FinnGen Biobank, and we used the release R11 with a total sample size of 136,188 individuals. ICD-10 codes were utilized to document diagnostic information for diseases. IVs data for PCa were obtained primarily from the Prostate Cancer Association Group for Investigation of Genomic Alterations in Cancer (PRACTICAL, https://practical.icr.ac.uk/), which synthesizes data from multiple studies to provide a reliable assessment of the risk associated with these genes. Other GWAS data for PCa were obtained from the IEU Open GWAS Project platform (https://gwas.mrcieu.ac.uk/datasets/), and all participants in the study were of European ancestry (S1Table). To strengthen the robustness of our findings and probe the potential causal relationship, we conducted a two-sample MR analysis utilizing independent, large-scale GWAS summary datasets. Genetic instruments for male infertility were sourced from the Finnish cohort (finn-b-N14_MALEINFERT), while PCa data were obtained from the UK Biobank (ukb-b-1392). Additionally, a multivariable MR analysis was conducted to control for potential confounding by genetically instrumented obesity, alcohol consumption, prostatitis, and type 2 diabetes, using datasets derived from the FinnGen database. A bidirectional two-sample MR analysis was performed between male infertility (finn-b-N14_MALEINFERT) and PSA (prot-a-1661) to preliminarily explore the direction of causality. Because we used only published research and pooled open access data, we did not need institutional review board approval for our analysis.

### Instrumental variable selection

The present MR analysis was based on summary-level GWAS data for male infertility and PCa. We initially selected IVs in accordance with the three fundamental assumptions of MR, with male infertility serving as the exposure factor. No IV were identified at the genome-wide significance threshold of *P* < 5 × 10⁻⁸. We therefore adopted a more lenient P-value threshold of *P* < 5 × 10⁻⁶ for the IVs of male infertility. To evaluate instrument strength, we calculated the cumulative R^2^ and single nucleotide polymorphism (SNP) specific F-statistics. In accordance with the conventional threshold for strong instruments, SNPs exhibiting F-statistic values < 10 were excluded to address potential weak instrument bias. While the F-statistic cannot directly correct for bias introduced by relaxing the significance threshold (*p* < 5 × 10⁻⁶), it serves as a critical tool for diagnosing and quantifying the risk and severity of weak instrument bias. This relaxed threshold was adopted cautiously only after a series of sensitivity analyses, including linkage disequilibrium clumping, leave-one-out analysis, MR-Egger regression, and tests for pleiotropy, were performed to ensure the robustness of the findings. SNPs were then independently screened. We set the explained variance of linkage disequilibrium coefficient (R^2^) at 0.001 and the genomic region width at 1000 kb to ensure the independence of each SNP and to minimize the influence of linkage disequilibrium on the results. We used NIH’s LDlink tool (https://ldlink.nih.gov/?tab=ldtrait/) to assess potential associations between the selected SNPs and confounding traits (e.g., obesity, smoking), removing any IVs that might introduce bias. In addition, we employed the FastTraitR package (v1.0.1) to systematically query significant associations between all candidate SNPs and a broad spectrum of traits. This step allowed us to identify and exclude IVs that exhibited genome-wide significant associations with age-related phenotypes, cardiovascular diseases, diabetes, and other potential confounders. SNPs associated with known confounders were excluded to satisfy the exclusion-restriction assumption of MR, ensuring that genetic variants function as valid IVs. In the reverse MR analysis evaluating the effect of PCa on male infertility, genome-wide significance thresholds were set at 5 × 10⁻⁸ and 5 × 10⁻⁶, respectively.

In this MR study, we reduced bias by checking three key MR assumptions. First, the IVs were significantly associated with the exposure. Second, the genetic variants were not associated with the outcome through confounding pathways. Third, the genetic variants did not directly affect the outcomes and were only indirectly associated with the outcomes through exposure-related factors. To assess sensitivity, we employed robust statistical methods (e.g., heterogeneity tests and pleiotropy assessments) to detect potential pleiotropic effects.

### Statistical analysis

Four MR analytical methods were utilized in this study: the inverse variance weighted (IVW) method served as the primary analytical framework, complemented by MR-Egger regression, the weighted median approach, and the maximum likelihood method. According to the IVs heterogeneity assessment, the fixed-effects model was employed for analysis. Under these circumstances, the model facilitates a relatively straightforward estimation of the weighted average causal effect predicated on IVs. The IVW method was chosen as the principal approach since it can generate consistent estimates under the assumption of balanced pleiotropy [11]. The complementary approaches were systematically implemented in concert to rigorously evaluate and validate the robustness of the findings. This multi-methodological strategy was designed to comprehensively assess the putative causal relationship between male infertility and PCa development. MR-Egger regression, which operates under the assumption that the strength of the IVs is independent of the direct effect, is robust to pleiotropy but remains sensitive to outliers and potential violations of the instrument strength independence assumption [12]. The weighted median approach computes the median of the ratio estimates from genetic variants. Under the assumption that there are linear and homogeneous associations between genetic variant loci and both exposure as well as outcome variables, all valid instrumental genetic variants are anticipated to produce consistent estimates of the same causal parameter. Although this approach shows a certain degree of robustness to outliers, it is still sensitive to the inclusion or exclusion of specific genetic variants [13]. The maximum likelihood method estimates the parameters that maximize the likelihood of the observed data. To assess heterogeneity, we utilized Cochran’s Q and Higgins *I*^2^ statistics, complemented by funnel plots for visual comparison of heterogeneity patterns [14]. Additionally, the reliability of the findings was evaluated using leave-one-out sensitivity analysis. All analyses were performed with the “TwoSampleMR” package (version 0.5.7) in R (version 4.4.0). The validity of the MR estimates was further assessed from three key perspectives using the “MRPRESSO” package: detection of horizontal pleiotropy, correction via outlier removal, and testing for significant differences in causal estimates before and after correction. For the multivariable MR analysis, causal effects were estimated using the IVW method implemented in the “MVMR” package.

### Genetic correlation analysis

To further investigate the genetic mechanisms connecting male infertility and PCa, we conducted an analysis using disequilibrium score regression (LDSC), a widely recognized tool for estimating heritability and genetic correlation from GWAS summary statistics (https://github.com/bulik/ldsc/). This method presents a notable advantage—it remains unaffected by sample overlap—allowing us to utilize the GWAS summary statistics from the Finnish database (R11 version) in our analysis of both diseases. In the results, the genetic correlation coefficient (rg) is employed to quantify the shared genetic architecture between traits, with values ranging from −1 to 1. It is worth noting that the closer the absolute value of rg is to 1, the stronger the genetic correlation between the traits. The associated p-value (rg_P) is obtained through a two-tailed test, and statistical significance is determined at *P* < 0.05.

### Summary data-based Mendelian randomization (SMR) analysis and Heterogeneity in Dependent Instruments (HEIDI) test

In this study, we used summary data from GWAS and cis-expression quantitative trait loci (cis-eQTL) to perform SMR analysis, with the aim of exploring potential pleiotropic associations between gene expression and PCa. Subsequently, the HEIDI test was carried out to assess whether the observed associations could be attributed to vertical pleiotropy rather than linkage disequilibrium with the dependent variable. This step is crucial for ensuring the validity of causal inferences in MR analyses. Regarding the SMR analysis, we set a significance threshold of 0.05. If the P-value was below this threshold, it was considered indicative of a potential association between expression levels and PCa risk. To assess the heterogeneity of IVs, we further performed HEIDI tests on the candidate genes identified through the SMR analysis. Only genes with P-values exceeding 0.05 in the HEIDI tests were retained.

## Results

### Instrumental variable selection

In this study, we analyzed the Finn-b-N14_MALEINFERT dataset from the FinnGen study and identified a total of 82 SNPs associated with male infertility, using a significance threshold of *P* < 10⁻⁶. To address linkage disequilibrium, we performed clumping analysis with a strict threshold of *r*^2^ < 0.001 and a window size of 10,000 kb. Additionally, we used the LDlink database to assess trait associations for each candidate SNP. This was done to control for the potential confounding effects of factors such as smoking and obesity on PCa etiology and to exclude the influence of other phenotypes that may be indirectly linked through IVs. Based on the core criteria of valid IVs, six SNPs were identified as IVs for male infertility (Table 1). The F-statistics for these SNPs ranged from 20.94 to 22.58, all exceeding the conventional threshold of 10, indicating a low likelihood of being weak instruments. For the reverse MR analysis, we utilized datasets from the IEU OpenGWAS project (ebi-a-GCST90018905). A total of 64 and 102 instrumental variables were identified at the genome-wide significance thresholds of *P* < 1 × 10⁻⁸ and *P* < 1 × 10⁻⁶, respectively. Following LD clumping (r^2^ < 0.001 within a 10,000 kb window) and the removal of SNPs linked to confounders or the outcome, a final set of 53 and 102 SNPs were retained for the respective analyses. The F-statistics for these SNPs ranged from 30.66 to 637.21, all exceeding the conventional threshold of 10, confirming their adequacy as strong IVs.

**Table 1:** Genetic variants used in the MR analysis

			Male infertility				Prostate cancer (ieu-b-85)		
SNP	EA	NEA	BETA	SE	P	Nearby gene	BETA	SE	P
rs115471009	G	A	− 1.0005	0.211671	2.28E-06	DNAH5	− 0.0325	0.0297	0.273833907
rs17428901	C	A	− 0.17015	0.0368096	3.79E-06	ANKRD46	0.0009	0.0085	0.915675696
rs434943	A	G	− 0.176626	0.037301	2.19E-06	MAGOH3P	0.0039	0.0091	0.668235142
rs57543282	G	C	− 2.85418	0.613088	3.23E-06	LINC02763	− 0.0069	0.0395	0.861328299
rs7046498	A	T	0.172617	0.0363293	2.02E-06	COL15A1	− 0.0012	0.009	0.893929767
rs79778261	T	C	− 0.613532	0.134083	4.74E-06	ZNF783	0.0034	0.0327	0.917188763

### Main Mendelian randomization results

**Fig. 2:**
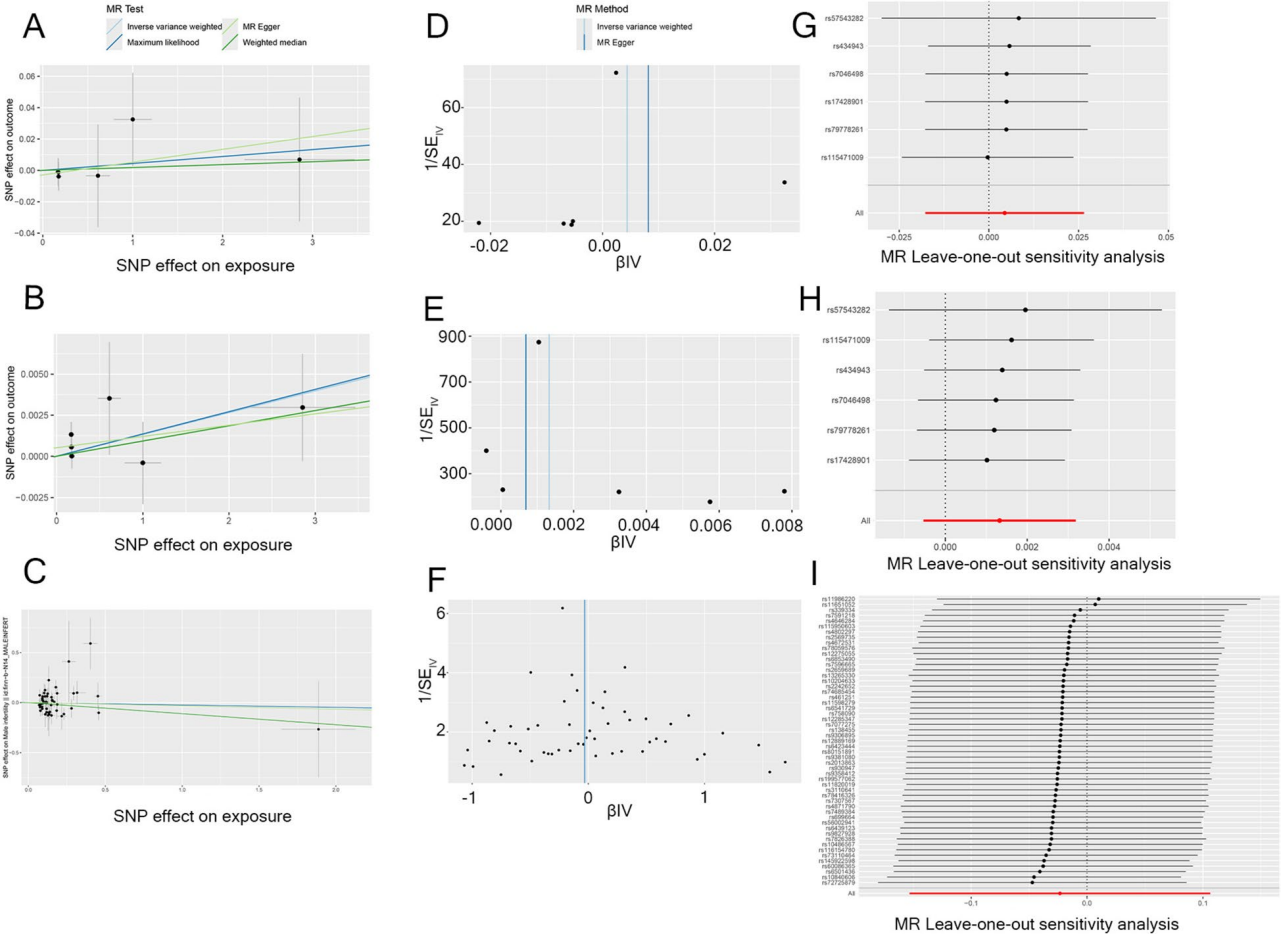
Evaluation of causal effects between male infertility and PCa using different MR methods. The forest plot shows the estimated causal effects and their 95% confidence intervals obtained using multiple MR methods. The results of the main analysis method, IVW, indicate that the gene-predicted male infertility is not associated with the risk of PCa (OR = 1.004, 95% CI 0.982–1.0269). The results of the weighted median method are consistent (OR = 1.002, 95% CI 0.975–1.03, *P* = 0.896), and the MR-Egger regression results are not significant (OR = 1.008, 95% CI 0.9815–1.036, *P* = 0.583), indicating that the main analysis results are robust. *PCa* prostate cancer

**Fig. 3:**
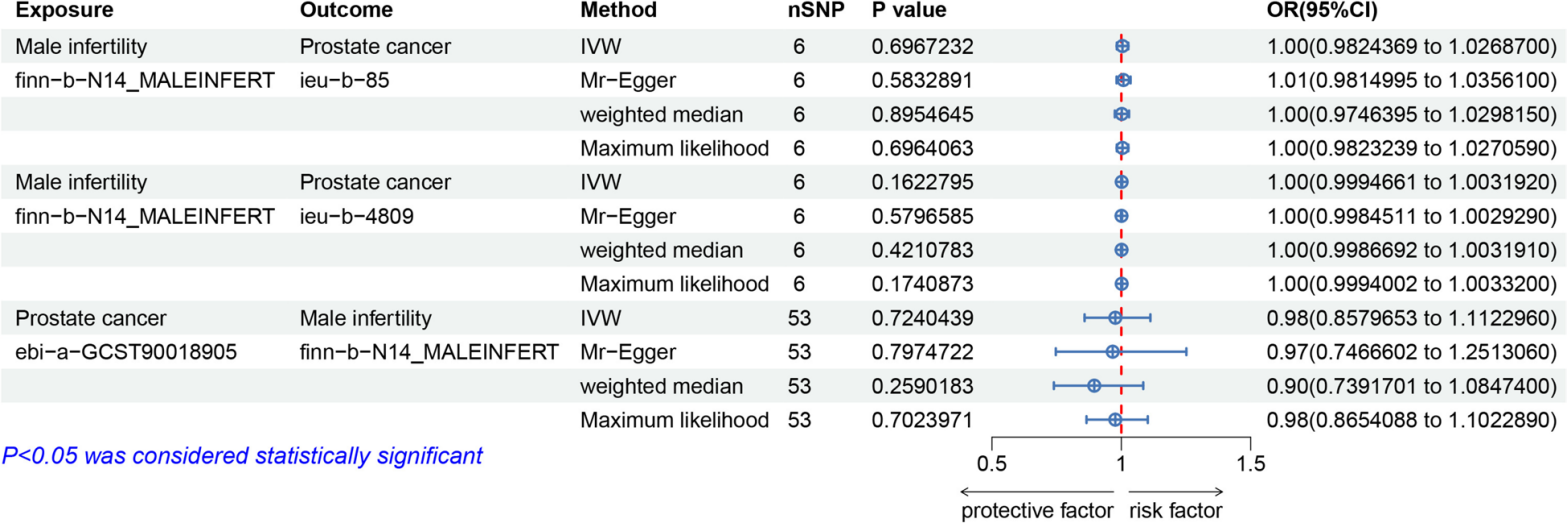
Robustness verification analysis of the causal relationship between male infertility and prostate cancer. **A**, **B**, **C** Scatter plots of MR analysis. The estimated values by the main IVW are as follows: OR = 1.004, 95% CI 0.982–1.0269, *P* = 0.697; OR = 1.001, 95% CI 0.999–1.003, *P* = 0.162; OR = 0.9769, 95% CI 0.858–1.112, *P* = 0.724. **D**, **E**, **F** Funnel plots of MR analysis. The effect size of the SNP is approximately symmetrical on both sides of the IVW estimated value. **G**, **H**, **I** Leave-one-out method of MR analysis. The graph indicates that the results of the entire MR analysis are not overly influenced by a specific instrumental variable. *MR* Mendelian Randomization, *OR* Odds Ratio, *CI* Confidence Interval, *IVW* Inverse Variance Weighting, *SNP* Single Nucleotide Polymorphism

**Fig. 4:**
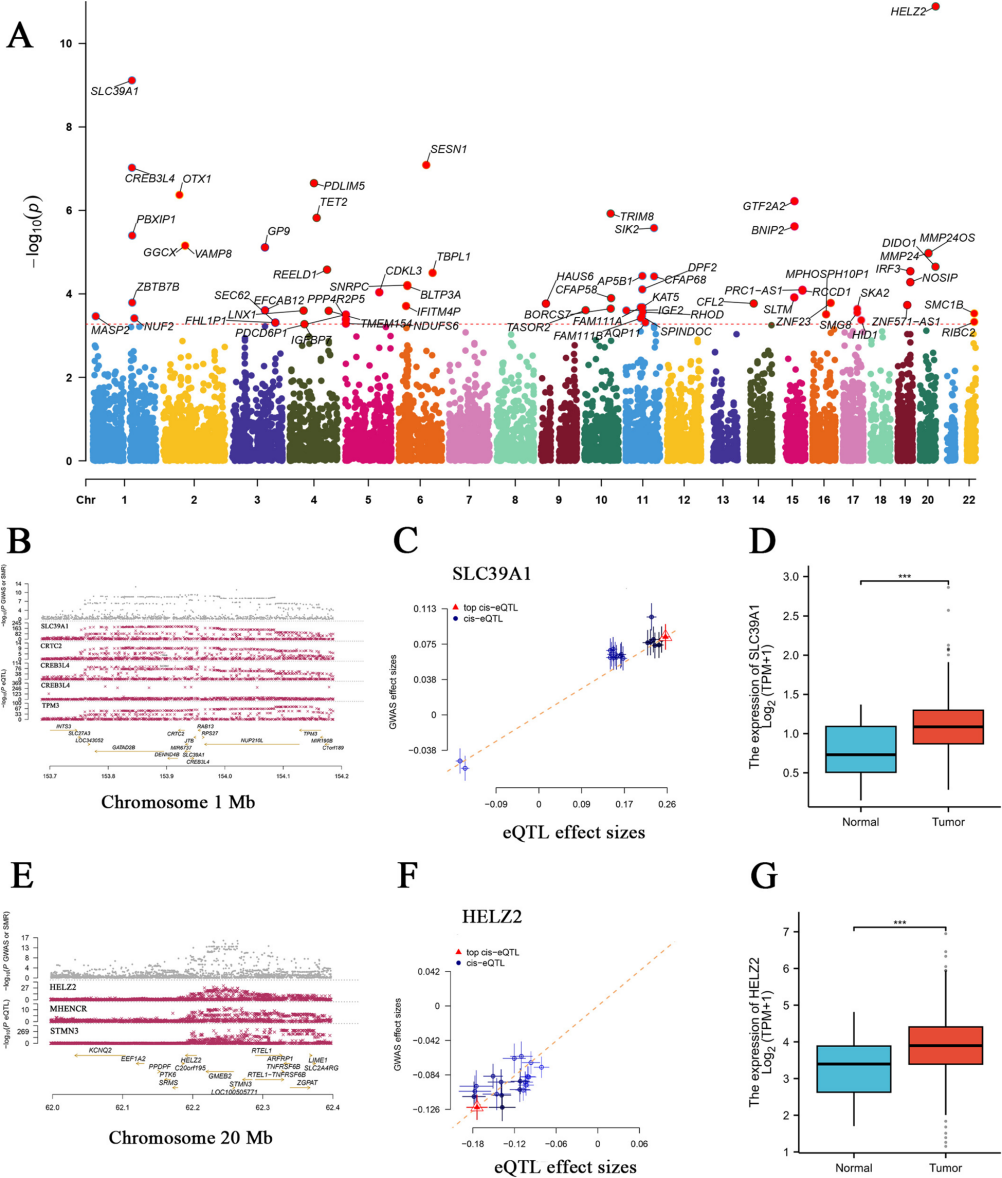
Screening and validation of causal genes related to PCa through SMR analysis. **A** Manhattan plot of the entire genome. The red line represents the significance threshold (*P* = 1.0 × 10–8). **B** Location map of the *SLC39A1* gene. **C** Scatter plot of the *SLC39A1* gene. The graph shows that the expression of the *SLC39A1* gene is positively correlated with the risk of PCa. **D** Verification of the expression level of the *SLC39A1* gene in PCa tissues (*n* = 501) and normal tissues (*n* = 52) using the TCGA database. The data show that the expression of *SLC39A1* is significantly upregulated in cancer tissues (*P* < 0.05). **E** Location map of the *HELZ2* gene. **F** Scatter plot of the *HELZ2* gene. The graph shows that the expression of the *HELZ2* gene is positively correlated with the risk of PCa. **G** Verification of the expression level of the *HELZ2* gene in PCa tissues (*n* = 501) and normal tissues (*n* = 52) using the TCGA database. The results show that the expression of *HELZ2* is significantly upregulated in cancer tissues (*P* < 0.05). SMR Summary data-based Mendelian Randomization, *TCGA* The Cancer Genome Atlas

The causal relationship between male infertility and the development of PCa was comprehensively evaluated through four distinct analytical methods. The primary analysis utilizing the IVW method demonstrated that genetically predicted male infertility, as represented by the instrument (finn-b-N14_MALEINFERT), was not associated with an elevated risk of PCa (ieu-b-85) (OR = 1.0044, 95% CI = 0.9824–1.0269, *P* = 0.697). Consistent findings were observed across the MR-Egger (OR = 1.0082, 95% CI = 0.9815–1.0356, *P* = 0.583), weighted median (OR = 1.0018, 95% CI = 0.9746–1.0298, *P* = 0.896), and maximum likelihood (OR = 1.0044, 95% CI = 0.9823–1.0270, *P* = 0.696) analyses( Table 2). Heterogeneity assessments employing Cochran’s Q statistic revealed no significant heterogeneity for either the MR-Egger (*P* = 0.9) or IVW methods (*P* = 0.935). Furthermore, the absence of horizontal pleiotropy was confirmed via the MR-Egger intercept test (*P* = 0.653). Sensitivity analyses incorporating leave-one-out evaluations for all significant SNPs corroborated the robustness of these findings, with no appreciable impact on the overall conclusions. In the reverse MR analysis using a genome-wide significance threshold (*P* < 5 × 10⁻⁸) for PCa (ebi-a-GCST90018905) as the exposure, the IVW method indicated that genetically predicted PCa was not a risk factor for male infertility (finn-b-N14_MALEINFERT) (OR = 0.98, 95% CI 0.86–1.11, *P* = 0.72). These findings were consistently supported by the MR-Egger (OR = 0.9666, 95% CI = 0.7467–1.2513, *P* = 0.798), weighted median (OR = 0.8954, 95% CI = 0.7392–1.0847, *P* = 0.259), and maximum likelihood (OR = 0.9767, 95% CI = 0.8654–1.1023, *P* = 0.702) analyses, as illustrated in Fig. 2. Cochran’s Q tests for both the MR-Egger (*P* = 0.169) and IVW (*P* = 0.194) analyses confirmed the absence of significant heterogeneity. Additionally, the MR-Egger intercept test (*P* = 0.926) provided no evidence of horizontal pleiotropy, and leave-one-out sensitivity analyses identified no significant outlier SNPs, as depicted in Fig. 3. Consistent results were obtained when using a more lenient significance threshold (*P* < 5 × 10⁻⁶) for the PCa instrumental variables (OR = 0.9769, 95% CI 0.8859–1.0358, *P* = 0.281). Furthermore, no significant heterogeneity or horizontal pleiotropy was detected for these instruments, as depicted in S4 Fig 4. Multivariable Mendelian randomization analysis, which included male infertility, obesity, alcohol consumption, prostatitis, and type 2 diabetes, indicated that only prostatitis was an independent risk factor for PCa after adjusting for these potential confounders (OR = 1.09, 95%CI 1.02–1.17, *P* = 0.001). No causal relationship was observed between PSA levels and male infertility in our Mendelian randomization analysis (IVW OR = 0.98, 95% CI 0.81–1.19, *P* = 0.847). Consistent null results were found in the reverse causality analysis (S4 Fig 4).

**Table 2:** Significant MR analysis results

Exposure	Outcome	SNPs	pleiotropy(*p*)	F(Median)	Cochrane’s Q(IVW)	MR method	P	OR	95%CI	
Male infertility	Prostate cancer	6	0.6529	22.01	0.935	IVW	0.697	1.0044	0.9824	1.0269
finn-b-N14_MALEINFERT	ieu-b-85					Mr-Egger	0.583	1.0082	0.9815	1.0356
						Weighted median	0.896	1.0018	0.9746	1.0298
						Maximum likelihood	0.696	1.0044	0.9823	1.0271
Male infertility	Prostate cancer	6	0.37	22.01	0.6158	IVW	0.162	1.0013	0.9995	1.0032
finn-b-N14_MALEINFERT	ieu-b-4809					Mr-Egger	0.58	1.0029	0.9998	1.0029
						Weighted median	0.421	1.0032	0.9987	1.0032
						Maximum likelihood	0.174	1.0014	0.9994	1.0033
Prostate cancer	Male infertility	53	0.926	56.62	0.1943	IVW	0.724	0.9769	0.858	1.1123
ebi-a-GCST90018905	finn-b-N14_MALEINFERT					Mr-Egger	0.798	0.9666	0.7467	1.2513
						Weighted median	0.259	0.8954	0.7392	1.0847
						Maximum likelihood	0.702	0.9767	0.8654	1.1023

### LDSC analysis

In our analysis leveraging genome-wide genetic variation data to assess genetic correlations via linkage disequilibrium, we found no significant genetic correlation between male infertility and PCa (Rg =  − 0.1102, *P* = 0.494). These results indicate a lack of compelling evidence for a shared genetic architecture between these two conditions .

### SMR analysis and gene expression analysis

To further evaluate the correlation between gene expression levels and PCa risk, we performed SMR analysis (*P* < 0.05) and HEIDI analysis (*P* > 0.05), using cis-eQTL datasets to robustly identify key genes associated with PCa risk (Fig. 4A). Subsequently, we applied the Wilcoxon rank sum test for differential expression analysis of the *HELZ2* and *SLC39A1* genes, which were previously identified. This analysis included 553 samples from the TCGA PCa dataset, comprising 52 normal and 501 tumor samples, aiming to validate their expression patterns at the transcriptomic level in clinical samples (Fig. 4D,G).

## Discussion

Our bidirectional MR analysis found no evidence supporting a causal relationship between male infertility and PCa in either direction, after careful adjustment for potential confounding factors. Multivariable MR analysis revealed that, after adjustment for several potential confounders, male infertility remained noncausally associated with PCa, whereas prostatitis constituted an independent risk factor. Although our MR analysis did not support a causal relationship, the persistent association observed in epidemiological studies suggests that these conditions may share underlying biological pathways operating at different stages of disease initiation and progression. For instance, the utility of the PCa biomarker PSA in screening for male infertility warrants investigation. Existing evidence indicates that seminal PSA levels correlate with sperm motility, implying a role for PSA in regulating male fertility. Furthermore, one study reported an association between PSA gene polymorphisms and infertility risk [15], while another found that infertile men exhibit significantly higher PSA levels than their fertile counterparts [16]. To formally assess the causal relationship between PSA levels and male infertility, we performed a bidirectional two-sample Mendelian randomization analysis. Our results did not provide robust evidence for a significant causal effect in either direction. Consequently, it is unlikely that PSA mediates the observed epidemiological association between infertility and PCa. Nonetheless, overlapping biological pathways governing PSA secretion and male reproductive function may still exist, meriting further investigation. Future studies should also leverage large-scale, high-quality cohorts to evaluate whether the levels or dynamic trajectories of specific PSA molecular forms could serve as diagnostic or screening tools for male infertility.

Although the genetic correlation between male infertility and PCa did not reach statistical significance, the observed negative estimate suggests a potential genetic overlap that may reflect an inhibitory relationship under specific genetic backgrounds. Further investigation into the shared genetic and environmental risk factors and underlying mechanisms of these two diseases may provide a basis for developing targeted screening strategies for male infertility. The androgen receptor (AR) plays a crucial role in the male reproductive system and various tissues. Its main mechanism is to regulate the expression of target genes by binding to androgens, thereby influencing physiological functions. Multiple meta-analyses have shown that AR polymorphisms are significantly associated with an increased risk of PCa [17–19]. Additionally, some studies have suggested that AR variations may also be related to isolated male infertility [20]. Spermatogenesis is exquisitely sensitive to DNA integrity, while prostate carcinogenesis is similarly driven by the accumulation of DNA damage and repair failure. Supporting this parallel, infertile men exhibit a markedly elevated frequency of sperm DNA breaks [21]. Additionally, alterations in DNA repair genes such as BRCA and ATM are frequently observed in PCa [22, 23]. Collectively, these findings suggest that the two conditions may share common underlying pathways, which converge on key biological processes including genetic damage repair, telomere maintenance, and chromosomal stability. In addition to shared etiological factors and physiological-functional connections, a recent study identified that all 25 genes associated with male factor infertility are known cancer progression factors, based on the classification by Vogelstein et al. [24]. Among these are specific polymorphisms of the AR, which influence susceptibility to PCa and are implicated in impaired spermatogenesis, particularly in cases of hereditary androgen insensitivity syndrome, a recognized cause of male infertility [25]. Another study revealed significantly altered genomic and transcriptomic patterns of some male infertility genes in PCa patients, indicating potential shared genetic mechanisms underlying both male infertility and PCa development [26]. At the transcriptional level, changes in circRNA expression levels have been associated with various urological disorders, ranging from weak spermatogenesis and nonobstructive azoospermia to PCa [27]. The majority of male infertility cases are linked to sperm quality, which depends on a complex anatomical system for production, storage, and transport. This process is regulated by the endocrine, immune, and nervous systems working together. Infertility can result from testicular dysfunction, endocrine disorders, lifestyle factors (like obesity and smoking), congenital anatomical issues, and aging. A meta-analysis of 13,077 men found obesity is a risk factor for male infertility [obesity (OR = 1.28, 95% CI = 1.06–1.55) and morbid obesity (OR = 2.04, 95% CI = 1.59–2.62)] [28]. For years, adipose tissue was thought to only store energy. But it also acts as an endocrine gland, affecting hormone levels. For example, excess visceral fat is tied to lower serum sex hormone-binding globulin, total and free testosterone, and inhibin B. The overproduction of cytochrome P450 aromatase in white adipose tissue boosts testosterone conversion to estradiol [29, 30], reducing the testosterone-to-estradiol ratio [31]. Elevated estrogen inhibits testosterone production by Leydig cells and spermatogenesis via Sertoli cells through negative feedback to the hypothalamic–pituitary–gonadal axis. Severe reductions in testosterone, which is essential for spermatogenesis, are linked to an increased risk of developing PCa [32]. Excessive visceral adipose tissue accumulation promotes the transport of free fatty acids to the liver, thereby reducing hepatic insulin clearance and increasing circulating insulin levels. Additionally, hypertrophic adipocyte apoptosis releases adipokines and inflammatory mediators. These factors significantly influence insulin signaling in insulin-responsive tissues, leading to systemic insulin resistance and hyperinsulinemia [33]. Elevated serum insulin and insulin resistance impair sperm motility by inhibiting spermatogenesis through increased nuclear and mitochondrial DNA damage, and they also reduce semen volume and sperm count [34]. The hyperinsulinemia caused by insulin resistance in obese patients also reduces the secretion of sex hormone-binding globulin by the liver. This leads to greater estrogenic activity and indirectly affects testosterone and spermatogenesis. Hyperinsulinemia is strongly associated with the development, progression, and aggressiveness of PCa [35]. As a potent growth factor that binds to cell membrane insulin receptors, insulin stimulates mitosis, resists apoptosis through protein kinase PKB/AKT-mediated signaling, and promotes DNA synthesis. It plays a crucial role in prostate carcinogenesis [36]. Elevated insulin receptor levels and increased subtypes in PCa support the possibility that PCa tissue can respond to changes in insulin levels [37]. Under obesity, adipocytes undergo progressive hypertrophy until their metabolic capacity is overwhelmed, leading to apoptosis. At this stage, adipocytes secrete a range of inflammatory factors and chemokines [38], which promote the proliferation and migration of monocyte–macrophages into adipose tissue. These infiltrating macrophages polarize into the M1 phenotype and activate the NF-κB pathway, thereby amplifying the production of proinflammatory factors. This creates a vicious cycle where obese patients gradually transition from a localized “low-grade chronic inflammatory state” to a systemic state [39, 40]. Inflammation induces aromatase expression, causing hormonal dysfunction, Leydig cell damage, and disruption of the blood-testosterone barrier, which leads to male infertility [41]. The systemic inflammatory state also generates reactive oxygen species and nitrogen compounds. These may directly activate proto-oncogenes or inactivate tumor suppressor genes, thereby promoting prostate carcinogenesis [42]. The release of interleukins and prostaglandins from tumor-associated macrophages (TAMs), which are recruited and generated locally by inflammation in the prostate gland, not only inhibits the antitumor immune response but also promotes tumor vascularization via the secretion of angiogenic factors such as Endothelin-2 and vascular endothelial growth factor (VEGF), thus further driving the progression of PCa [43]. In addition to obesity and a series of factors derived from inflammation providing a common potential pathogenic basis for these two diseases, more and more evidence indicates that oxidative stress, endocrine disorders, and telomere shortening may simultaneously damage spermatogenesis and initiate prostate carcinogenesis. Oxidative stress is defined as the pathological imbalance between the production of reactive oxygen species (ROS) and the antioxidant defense. ROS are highly reactive oxygen metabolites containing one or more unpaired electrons, and in a physiological balance state, they are crucial for processes such as sperm capacitation, acrosome reaction, and sperm–egg fusion. However, pathological imbalances of ROS caused by factors such as varicocele or infection can interfere with the normal development and function of sperm, thereby damaging male fertility [44]. Moreover, due to the rich content of polyunsaturated fatty acids in the sperm plasma membrane, it is highly susceptible to attack by ROS. Lipid peroxidation of the plasma membrane can generate a series of toxic substances, such as malondialdehyde (MDA) and 4-hydroxyneonaldehyde, which directly cause damage to the structure and function of sperm [45, 46].Epidemiological, experimental, and clinical studies have all confirmed that oxidative stress plays a significant role in the occurrence and development of PCa [47]. Research indicates that reactive oxygen species have a dual regulatory function in PCa: on one hand, ROS promotes tumor cell proliferation by activating signal pathways such as MAPK; on the other hand, it exerts an anti-apoptotic effect by regulating the mitochondrial apoptosis pathway and influencing caspase activity. This dual regulatory mechanism is crucial for the survival of PCa cells and helps them acquire clonal advantages during evolution [48]. Hormonal imbalance serves as the core link connecting the two diseases. The hypothalamus–pituitary–gonadal axis, as the key regulatory system for male reproductive endocrine, influences the occurrence and progression of PCa by regulating androgen levels. Currently, the main treatment for metastatic PCa is androgen deprivation therapy. The common approach involves using gonadotropin-releasing hormone agonists/antagonists to intervene in the HPG axis function, achieving drug castration [49]. At the same time, the luteinizing hormone, follicle-stimulating hormone, and testosterone regulated by the HPG axis play indispensable roles in Sertoli cells proliferation, testicular development, and function maintenance, as well as spermatogenesis. Dysfunction of this axis can interfere with the sperm production process, thereby leading to endocrine infertility [50]. Studies have shown that endocrine disruptors not only may increase the risk of cancer by targeting the prostate [51] but also can cause a decline in semen quality, manifested as abnormal sperm concentration, motility, and morphology [52]. Telomeres are highly conserved, noncoding, repetitive DNA sequences located at the ends of chromosomes. Their main function is to maintain chromosome integrity and genomic stability. Telomere abnormalities are one of the earliest molecular events in the development of PCa and continue to play a role in tumor progression. Studies have shown that significant telomere shortening can be observed in PCa cells and high-grade prostate intraepithelial neoplasia tissues [53]. During reproduction, telomeres participate in regulating reproductive lifespan by mediating chromosome pairing and homologous recombination during gamete formation. Shortening of telomere length in germ cells can lead to meiotic arrest and chromosome segregation errors, and subsequently increase in aneuploid germ cells and germ cell apoptosis [54].

This study employed the SMR method to identify a group of genes expressed in blood tissues that are associated with the risk of PCa. Among them, the expression levels of SLC39A1 and HELZ2 genes showed particularly strong statistical evidence for the causal association with the risk of PCa. The SLC39A1 protein acts as a zinc transporter to mediate the absorption and accumulation of zinc in prostate cells [55]. Multiple studies have shown that there are significant differences in zinc accumulation between normal prostate cells and cancer cells [56–58]. Clinical and experimental evidence has confirmed that the changes in SLC39A11 and zinc are related to the transformation of normal epithelial cells of the prostate gland to malignant cells [59]. A gene expression prediction model established using the whole transcriptome association study of blood tissues for PCa cases and controls suggested that the expression level of the SLC39A1 gene in PCa patients was significantly higher than that in the control group [60]. HELZ2 is a functionally complex helicase that can work in synergy with ribonuclease and can be induced by interferons, indicating its role in cellular defense. The three mutations found in cancer patients (D1601N, S1920L, and R1923L) eliminate the RNase activity of HELZ2, suggesting that they may promote tumor development by weakening the immune system [61]. Studies have shown that this gene can induce tumor formation by regulating the K63 polyubiquitination of c-Myc[62]. In another study based on RNA-binding proteins, HELZ2 was confirmed as a core biomarker promoting biochemical recurrence of PCa [63]. Although these genes inferred based on genetic data provide new insights into the potential mechanisms of PCa occurrence, more comprehensive functional experiments are still needed to further clarify the specific mechanisms of these genes in the occurrence of PCa. The present study possesses several notable strengths. First, the participants in the GWAS dataset for exposure and outcomes were all of European descent, which enhances the reliability of the results. Second, the application of MR showed that there was no reverse causality between the two variables. Furthermore, the use of different databases for validation strengthened the robustness of the results. However, this study also has some limitations. First, because all participants in this study were of European ancestry, the findings should not be extrapolated to other populations without additional validation. As GWAS datasets continue to expand and become increasingly refined, future investigations are expected to encompass genetically and environmentally diverse cohorts—particularly those bearing a disproportionate burden of PCa. Such studies will be essential for validating and extending the present findings across populations. Secondly, as a result of the inherent limitations of the GWAS data, detailed analysis of the relationship between male infertility subtypes and PCa histological subtypes was not feasible. With the ongoing advancements of genetic epidemiology and the anticipated expansion of more detailed and extensive GWAS datasets in the future, our understanding of the molecular mechanisms underlying these two diseases will be significantly enhanced. These developments will also improve molecular classification strategies as well as facilitate the identification of novel therapeutic targets. Ultimately, these advances will serve as a solid foundation for the development of precision medicine interventions in the fields of male reproductive health and urological oncology.

## Conclusion

This MR study found no evidence to support a causal relationship between male infertility and the risk of PCa. This null association remained consistent across univariable, reverse, and multivariable MR analyses, and was robust to a suite of sensitivity assessments.

## Supplementary Information

Supplementary material 1. S1 Table. Data resources used in the current study. 

Supplementary material 2. S2 Table. Information on the data sources. 

Supplementary material 3. S3 Table. The results of the SMR analysis for prostate cancer. 

Supplementary material 4. S4 Table. S4 Fig. Expression of the* SLC39A1* and *HELZ2* genes in the TCGA database.

Supplementary material 5.

## Data Availability

The GWAS summary data used in this study are publicly available. The data on male sterility were obtained from the FinnGen database (accessed via https://www.finngen.fi/en/access_results). The prostate GWAS data were retrieved from the IEU Open GWAS project (https://gwas.mrcieu.ac.uk/datasets/). Specifically, the datasets used were identified by searching for “ieu-b-85” and “ieu-b-4809” in the IEU Open GWAS database. Further inquiries can be directed to the corresponding authors.

## Abbreviations

PCa: Prostate cancer
GWAS: Genome-wide association study
IVW: Inverse variance weighted
LDSC: Linkage disequilibrium score regression
SMR: Summary data-based Mendelian randomization
IVs: Instrumental variables
RCTs: Randomized controlled trials
cis-eQTL: Cis-expression quantitative trait loci
TAMs: Tumor-associated macrophages
VEGF: Vascular endothelial growth factor
