# Supplementary material for: Male infertility and the risk of developing prostate cancer: a bidirectional two-sample Mendelian randomization study
Source: Eur J Med Res. 2025 Dec 1;31:21. doi: 10.1186/s40001-025-03461-y (PMC12777069; doi:10.1186/s40001-025-03461-y)

**Figure 1. Distribution of F-statistics for SNPs related to male infertility.** This distribution shows the strength of the six instrumental variables used in the Mendelian randomization analysis. All SNPs exceeded the recommended threshold (F > 10, as indicated by the dotted line), and the range of F-statistics was from 20.94 to 22.58.

**
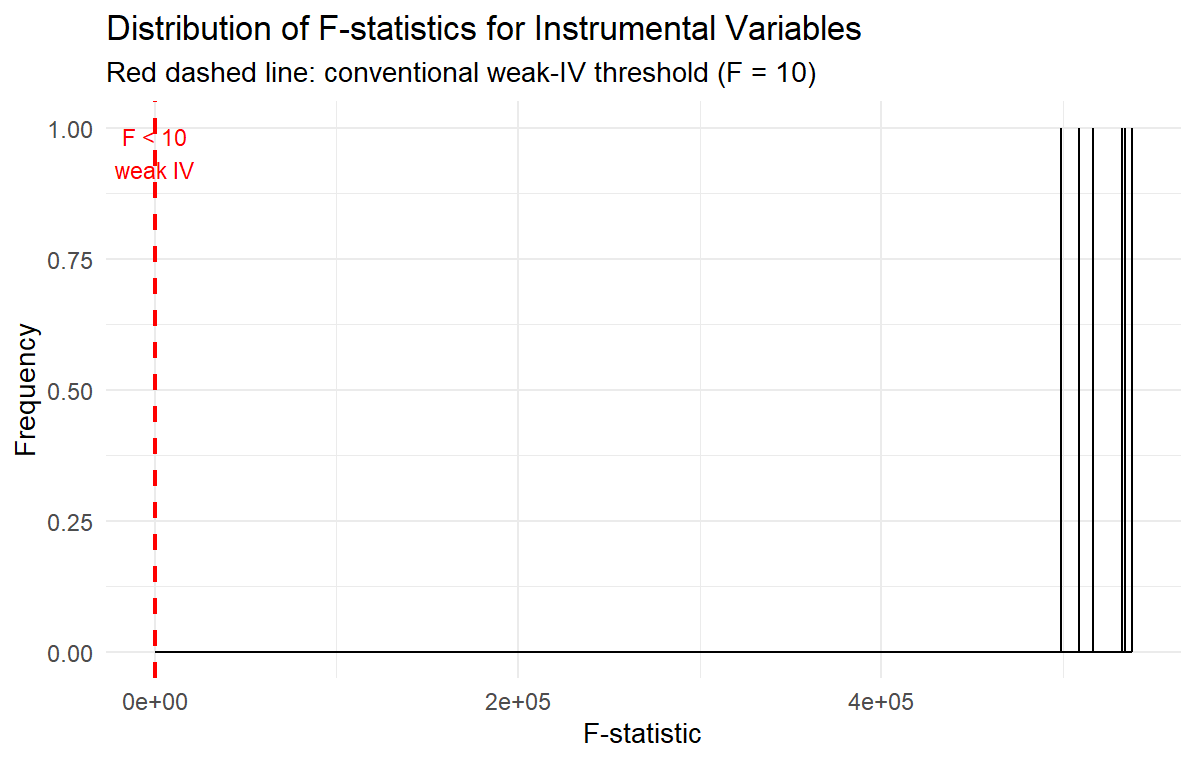
**

**Figure 2. Expression of the *SLC39A1* and *HELZ2* genes in the TCGA database.** (A,B) High expression of the *SLC39A1* gene significantly shortened the progression-free survival period (P < 0.05), suggesting that it could serve as a potential biomarker for predicting the risk of disease progression. The AUC was 0.696, indicating a moderate level of predictive ability. (C,D) The progression-free survival period of the high-expression group of the *HELZ2* gene was slightly shorter than that of the low-expression group, with a faster curve decline and a higher AUC (0.740), indicating slightly stronger predictive ability than *SLC39A1*. However, the Log-rank test showed that the difference was not statistically significant (P = 0.33), so it cannot be concluded that the expression level of this gene is significantly associated with PFI. Abbreviations: AUC, Area Under the Curve; PFI, Progress Free Interval.


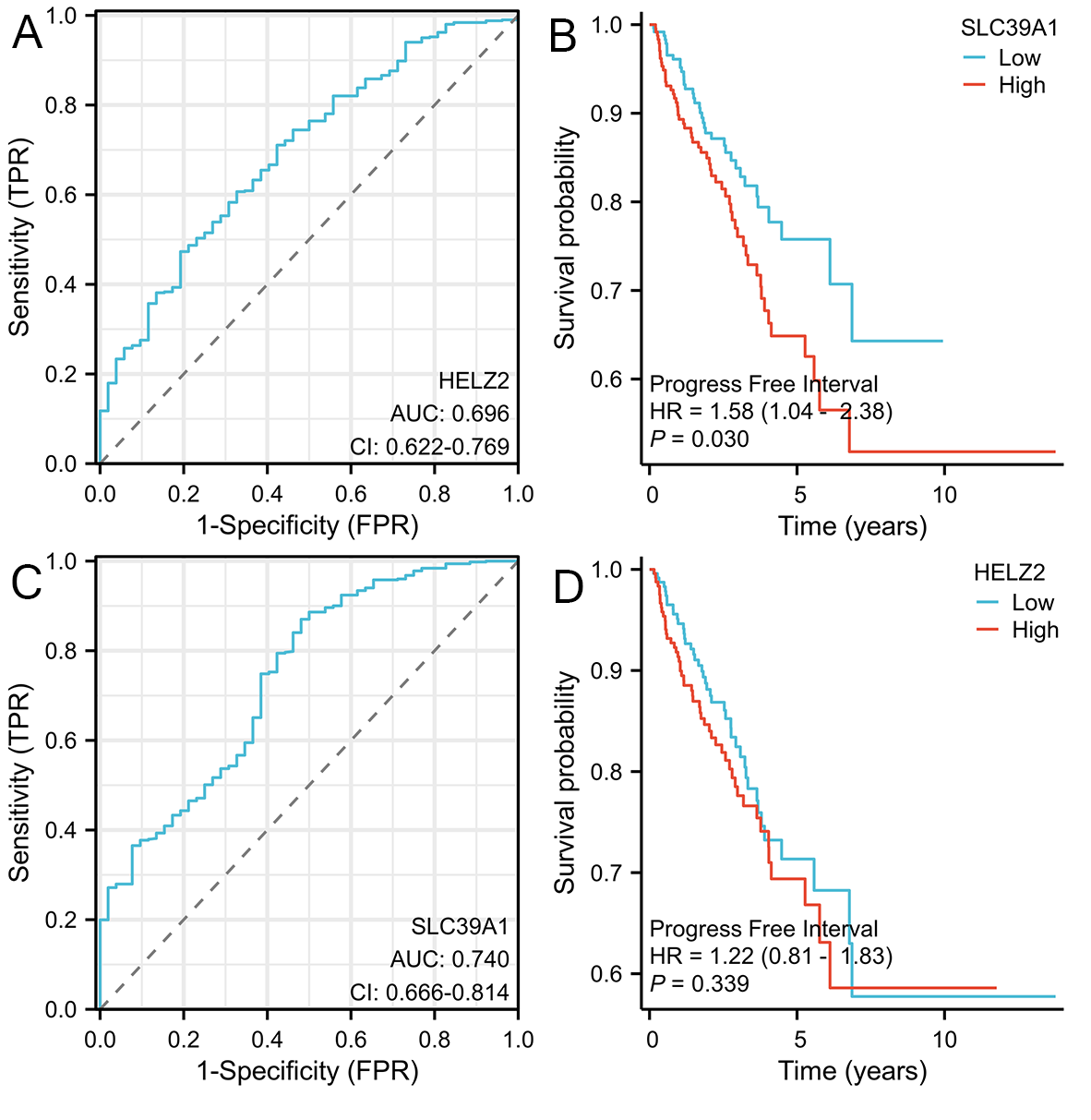


**Figure 3. **Forest plots for MR analyses.**** Results are shown from analyses of PCa and male infertility using varied MR methods, including those applied to the UK Biobank (ukb-b-1392) and FinnGen (finngen_R11_N14_MALEINFERT) datasets, MR under a relaxed exposure threshold (P < 5×10⁻⁶) for PCa, and bidirectional two-sample MR between PSA and male infertility. Abbreviations: **MR, Mendelian randomization;** PCa, Prostate cancer; PSA, Prostate-Specific Antigen.

**
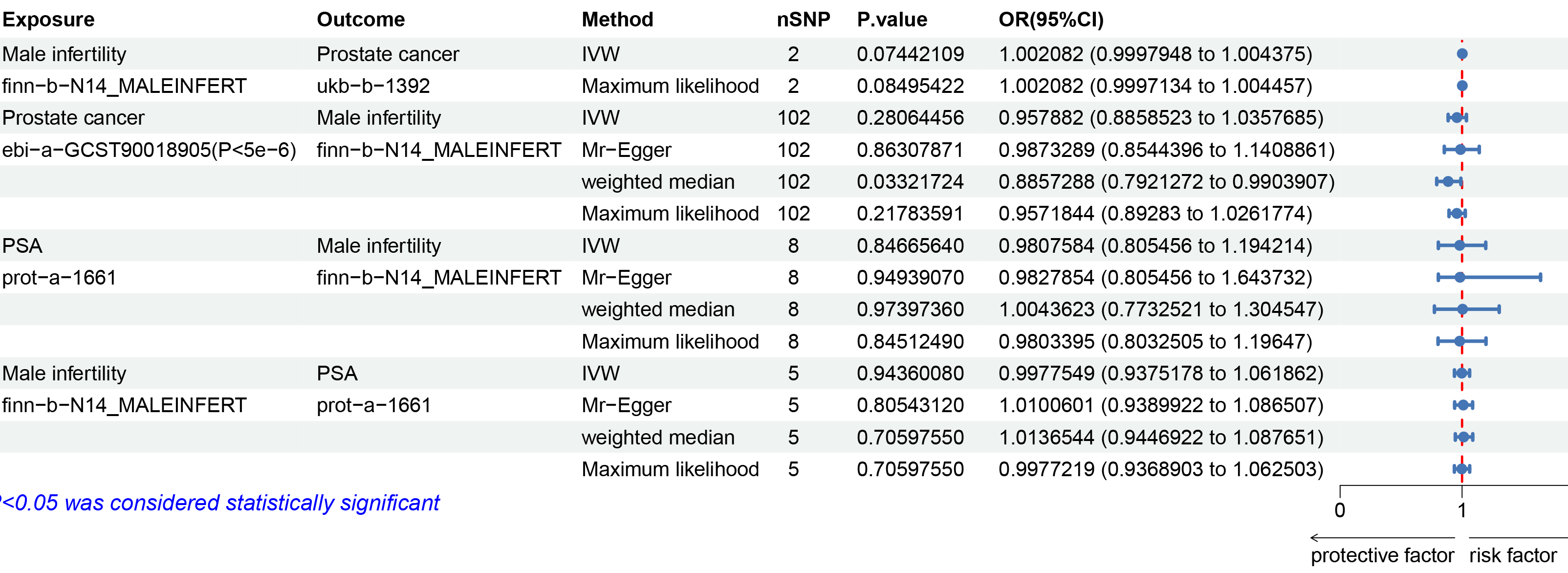
**

**Figure 4. **Sensitivity analyses for .**** (A-C) Scatter, funnel, and leave-one-out plots for the two-sample MR of PCa on male infertility, using a genome-wide significance threshold of P < 5×10⁻⁶ for instrumental variable selection. (D-F) Corresponding plots for the two-sample MR of PSA on male infertility. (G-I) Reverse MR analyses assessing the effect of male infertility on PSA levels. Abbreviations: MR, **Mendelian randomization; PCa,** prostate cancer; PSA, **Prostate-Specific Antigen.**

**
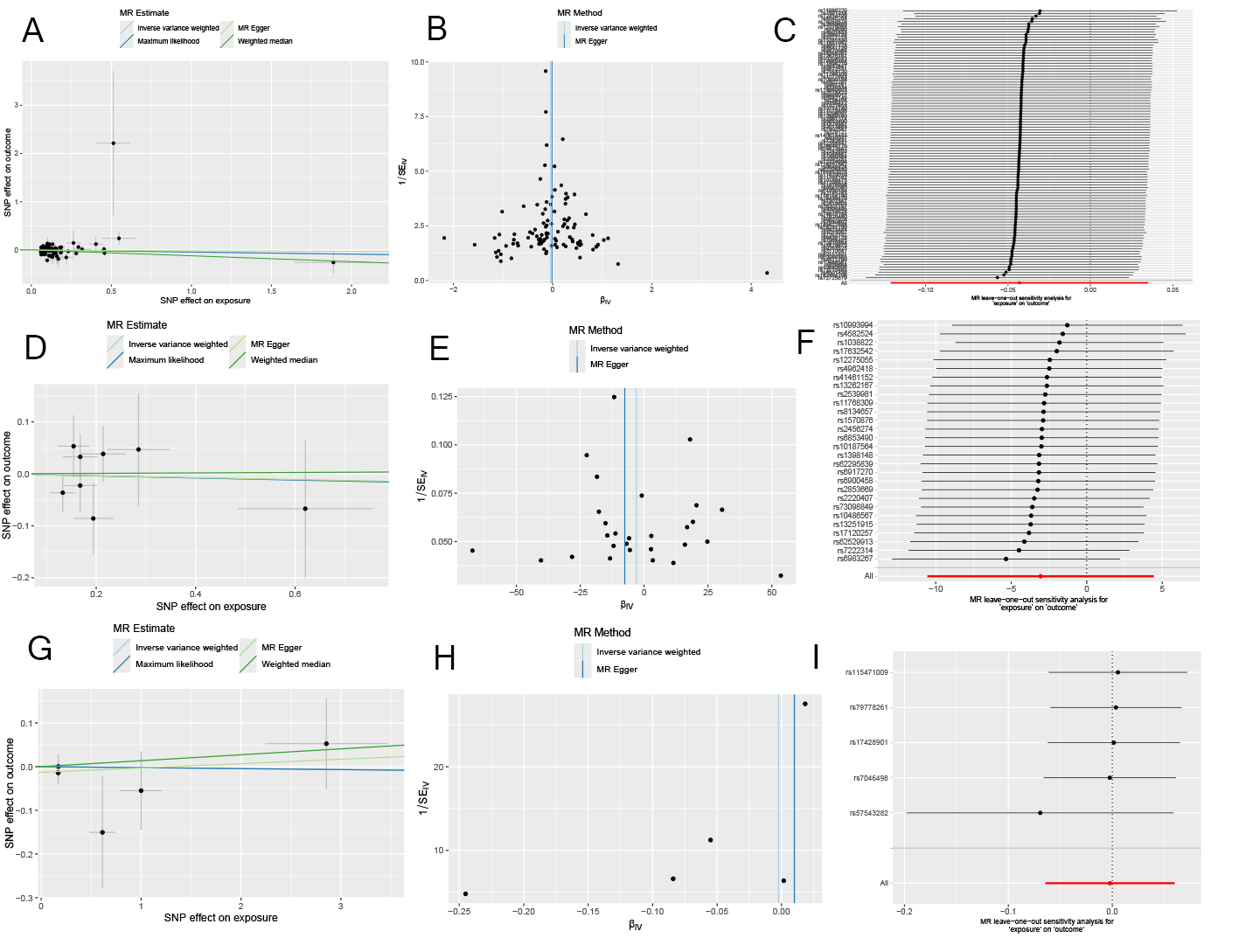
**

**Figure 5.Forest plot of multivariate Mendelian randomization results.** OR, Odds Ratio; CI ,confidence .interval.


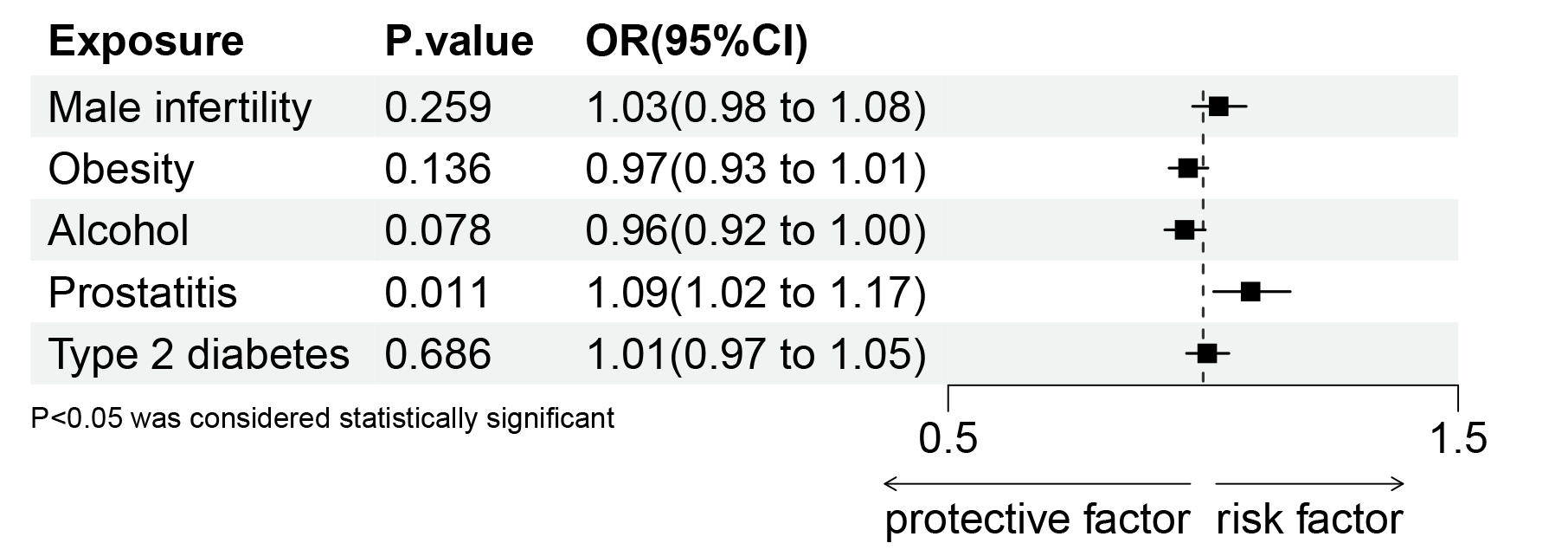

Supplement: Supplementary file 5 — Supplementary material 5. [file 40001_2025_3461_MOESM5_ESM.docx]
